# Effects of Great Gerbil Disturbance on Photosynthetic Characteristics and Nutrient Status of *Haloxylon ammodendron*

**DOI:** 10.3390/plants13111457

**Published:** 2024-05-24

**Authors:** Jinshun Shi, Xingming Hao, Zhongke Wang, Meng Jiang, Mengwen Peng, Jiaqi Bai, Li Zhuang

**Affiliations:** 1 College of Life Sciences, Shihezi University, Shihezi 832000, China; a15202542403@163.com (J.S.); 13897717695@163.com (Z.W.); jiangmengzwx@163.com (M.J.); 13369723327@163.com (M.P.); baijiaqi@stu.shzu.edu.cn (J.B.); 2 Xinjiang Institute of Ecology and Geography, Chinese Academy of Sciences, Wulumuqi 830011, China; 20222006035@stu.shzu.edu.cn

**Keywords:** *Haloxylon ammodendron*, *Rhombomys opimus*, gas exchange parameters, chlorophyll fluorescence parameters, nutrients

## Abstract

Rodents, such as those that feed on plants and nest in plant roots, can significantly affect the growth and development of desert plants. The aim of this study was to investigate the effects of *Rhombomys opimus* disturbance on the photosynthetic characteristics and nutrient status of *Haloxylon ammodendron* at different growth stages in the Gurbantunggut Desert. The effects of great gerbil disturbance on the photosynthetic characteristics of *H. ammodendron* at different growth stages were investigated by measuring the gas exchange parameters, instantaneous water use efficiency, and chlorophyll fluorescence parameters of *H. ammodendron* at different ages (young, middle, and adult) under the disturbance of great gerbils. The soil nutrients in the assimilated branches and rhizosphere of *H. ammodendron* at different growth stages were tracked to reveal the relationship between the *H. ammodendron* nutrient content and gerbil disturbance. The results showed that great gerbil disturbance decreased the organic carbon content in the rhizosphere soil of adult *H. ammodendron* and increased the total nitrogen content in the rhizosphere soil and the nitrogen and potassium contents in the assimilated branches at each growth stage. The net photosynthetic rate and instantaneous water use efficiency of *H. ammodendron* decreased at each growth stage, and the maximum photochemical efficiency and non-photochemical quenching parameters of the young *H. ammodendron* decreased. However, the actual photochemical efficiency and photochemical parameters of the middle *H. ammodendron* increased. It was concluded that the disturbance of great gerbils decreased the photosynthetic capacity of *H. ammodendron* and increased the content of total nitrogen in the soil and nitrogen and potassium in the plant. This study revealed that the Gurbantunggut Desert great gerbil and *H. ammodendron* do not have a simple predation relationship. It laid a foundation for the study of the moderate disturbance threshold and better use of the mutually beneficial relationship between the two.

## 1. Introduction

Rodents are among the most important phytophagous mammals, and their feeding and nesting behaviors have varying degrees of influence on plants [1]. Owing to their strong reproductive ability and large numbers, rodents have caused varying degrees of harm to human production and life in terms of disease transmission and agriculture, animal husbandry, and forestry [2]. The great gerbil (*Rhombomys opimus*) is a highly social rodent in the desert areas of northwest China [3] and feeds on desert plants such as *Haloxylon ammodendron* and *Tamarix hispida,* particularly the phloem and branches of *H. ammodendron* [4,5]. The density of rat holes exceeded 18.8% in some desert forests on the southern margin of the Gurbantunggut Desert in Xinjiang, indicating that these areas have been transformed into high-density rat infestation areas [6].

*H. ammodendron* is a shrub or small tree belonging to the genus *H. ammodendron*. It is one of the main types of desert vegetation and plays an important role in maintaining the regional ecological balance. It has remarkable drought and cold resistance and saline–alkali tolerance, and it is an established species in desert ecosystems [7,8]. It grows most vigorously in July and August [9]. In recent years, several studies have focused on the influence of gerbils on the growth and development of *H. ammodendron* [10] and have shown that the perturbation behaviors of great gerbils, especially their feeding and nesting behaviors, have significant negative effects on the growth and distribution of *H. ammodendron*. In addition, some studies have indicated that mild gerbil disturbances may contribute to the growth of *H. ammodendron*, whereas severe disturbances may limit its growth [5].

Photosynthesis is the physiological basis of plant growth and development and is at the core of the energy acquisition and conversion processes [11,12]. By optimizing the photosynthetic characteristics, the nonstructural carbohydrate content in its assimilated branches can be maintained in a stable state during drought, providing the necessary conditions for the survival and development of this species in harsh environments [13]. Previous studies have shown that photosynthetic efficiency under different environmental conditions, especially extreme drought conditions, is an important indicator of the survival ability of desert plants and their ability to adapt to external environmental changes [14,15,16]. In addition, when plants are damaged, their photosynthetic physiology may undergo adaptive adjustments to reduce the negative effects of damage to photosynthesis. This adaptive adjustment includes adjusting the gas exchange and chlorophyll fluorescence parameters to maintain or enhance the viability and resilience of damaged plants [17,18,19]. As a key technology for studying a plant’s photosynthetic physiology, chlorophyll fluorescence can be used to directly and effectively monitor the physiological and ecological processes of vegetation and environmental stress [20,21]. This technology is mainly used to study the physiological responses of plants under stress conditions and reveal the mechanisms of absorption, conduction, dissipation, and distribution of light energy in plant photosystems [22,23], which are closely related to photosynthesis in plants under natural conditions. By studying the photosynthetic characteristics of plants under different environmental conditions, we can understand how plants respond to changes in external conditions by adjusting their photosynthetic physiological processes, thus revealing the complex mechanisms of adaptation to environmental stress.

The total amount and availability of soil nutrients are important indicators of soil fertility and have significant effects on plant growth and development [24,25]. Soil nutrient status plays an important role in the survival and adaptation of desert plants that live in extreme environments, such as *H. ammodendron*, to external adverse factors [26,27]. Carbon, nitrogen, phosphorus, potassium, and other elements in plant nutrients play important roles in a plant’s physiological metabolism, and their contents and stoichiometric ratios reflect the absorption and utilization efficiency of nutrients by desert plants and their survival strategies to adapt to desert environments [25]. Through the mutual regulation of various elements, plants achieve stability in their biostoichiometric characteristics during growth and development, thus promoting stable growth and enhancing their stress resistance [28]. Therefore, it is of great scientific and application prospect value to study the nutrient uptake in the branches of *H. ammodendron* to understand its survival and adaptation mechanisms in desert environments.

The effects of gerbil disturbances on the photosynthetic characteristics of *H. ammodendron* have not yet been reported. Therefore, the purpose of this study was to investigate the effects of gerbil interference on photosynthesis, especially changes in the photosynthetic characteristics and nutrient status of *H. ammodendron* at different growth stages. By comparing the photosynthetic characteristics, nutrient accumulation, and rhizosphere soil nutrient changes of the assimilated branches of *H. ammodendron* that fed and those that did not feed, the aim was to reveal the physiological and ecological mechanisms of *H. ammodendron* in response to biological disturbances and provide a scientific basis for predicting and evaluating the effects of biological disturbances on desert ecosystems.

## 2. Methods and Materials

### 2.1. Study Area

The research area was located on the southern edge of the Gurbantunggut Desert (44°15′ N–46°50′ N, 84°50′ E–91°20′ E) in the Xinjiang Uygur Autonomous Region in China, with an average elevation of 300–600 m and which is the second-largest desert in China. The interior of the desert is dominated by fixed or semi-fixed dunes, which account for approximately 97% of the total desert area. *H. ammodendron* and *Haloxylon persicum* are the dominant species in the plant community of the Gurbantungut Desert, and the typical rodents are great gerbils and *Meriones meridianus* Pallas. The most common species are *Meriones tamariscinus*, *Meriones libycus*, and *Dipodidae* spp.

### 2.2. Experimental Design

Field surveys and sample collection were conducted at the study site in August 2023. In this study, three quadrats (50 m × 50 m) were set up in the rat hole area and the control area (no significant gerbil activity). The number of effective rat holes in each plot was kept at 6–9 according to the method of detecting and sealing burrows (The rat holes were gently blocked with sand or dead grass, and the rat holes that were open after 48 h were effective holes.) to ensure the uniformity of sampling in the rat hole area. Three sub-quadrats (10 × 10 m) with different growth stages were set in each plot, and the distance between each sub-quadrat exceeded 20 m. Each plot included at least 30 plants. According to the classification of Zhang et al. [29] and Luo et al. [30], *H. ammodendron* was divided into three growth stages based on plant height: young age (30–100 cm), middle age (100–200 cm), and adult age (>200 cm).

During the investigation, it was found that the burrows of great gerbils were generally 60 cm away from the surface [31,32]. Therefore, rhizosphere soil samples of *H. ammodendron* at different growth stages were collected in this study. In order to maintain uniformity, we collected the soil samples, which were located at depths 0–20, 20–40, and 40–60 cm below the surface. Then, the soil at the three depths was evenly mixed as soil samples to determine the soil nutrients. Three rhizosphere soil samples and their assimilated branches were collected from each subquadrat. The subquadrat soil samples were uniformly mixed to obtain composite samples of soil and assimilated branches. Soil and anabolic branch samples were collected using the same method as that used for the control area.

The collected soil and plant samples were brought back to the laboratory, and the soil samples were naturally air-dried, ground, and sifted through a 0.2 mm sieve for analysis. The samples were dried in an oven at 90 °C for 24 h to a constant weight, treated with a crusher, and then sieved through a 40 mesh screen for nutrient determination.

### 2.3. Determination of Gas Exchange Parameters

The gas exchange parameters of *H. ammodendron* were measured using an Li-6400 (LI-COR Inc., Lincoln, NE, USA) portable photosynthesis apparatus under continuous, clear weather in August 2023. The test subjects included *H. ammodendron* at each growth stage (young, middle-aged, and adult) in the areas with rat holes and the control area. Three plants with relatively uniform growth were selected for each growth stage. Healthy assimilated branches in the middle of the plants were selected for determination. Three values were read each time. The average value of each parameter was considered the average value. The measurements were performed every 2.5 h from 8:00 a.m. to 8:30 p.m. The measured parameters were the net photosynthetic rate (Pn), transpiration rate (Tr), intercellular CO_2_ concentration (Ci), stomatal conductance (Gs), and instantaneous water use efficiency (WUE = Pn/Tr) of the leaves.

### 2.4. Determination of Chlorophyll Fluorescence Parameters

The chlorophyll fluorescence parameters of *H. ammodendron* were determined using a DUAL-PAM-100 dual-channel chlorophyll fluorescence measuring system (Walz, Effeltrich, Germany) in clear and windless weather in August 2023. The measurements began at 10:00 a.m. Healthy, growing branches were selected, and dark adaptation clips were used for 25 min of dark adaptation treatment before the chlorophyll fluorescence parameters were determined. The measured parameters included the maximum photochemical efficiency (Fv/Fm), actual photochemical efficiency (Y(II)), photochemical quenching (qP), and non-photochemical quenching (NPQ).

### 2.5. Determination of Nutrients

The soil’s organic carbon (SOC), total nitrogen (TN), total phosphorus (TP), and total potassium (TK) were determined using potassium dichromate volumetric external heating, perchloric acid-sulfuric acid digestion, acid-soluble molybdenum-antimony resistance colorimetric, and acid-soluble atomic absorption methods [33]. The carbon (C), nitrogen (N), phosphorus (P), and potassium (K) contents of the assimilated branch nutrients were also determined using the corresponding methods.

### 2.6. Statistical Analysis

Microsoft Excel was used to calculate and organize the data, and an independent sample *t*-test was used to compare the effects of gerbil disturbance on *H. ammodendron*’s branch assimilation, soil nutrients, and chlorophyll fluorescence parameters. A post hoc Duncan’s multiple comparison test was used to examine the differences in the nutrient and chlorophyll fluorescence parameters in the assimilated branches and soil at different growth stages. A general linear model (GLM) was used to analyze the effects of the growth stage, gerbils, and their interactions on the soil and plant nutrients. The relationship between the nutrient and photosynthetic parameters was analyzed using Pearson’s correlation analysis. The significance level was set at *p* < 0.05. SPSS 23 software (SPSS Inc., Chicago, IL, USA) was used for data analysis. Graphics were drawn using OriginPro 2024 (OriginLab Corp., Northampton, MA, USA).

## 3. Results

### 3.1. Effects of Great Gerbil Disturbance on Gas Exchange Parameters and Instantaneous Water Utilization of H. ammodendron Assimilated Branches

**Figure 1:**
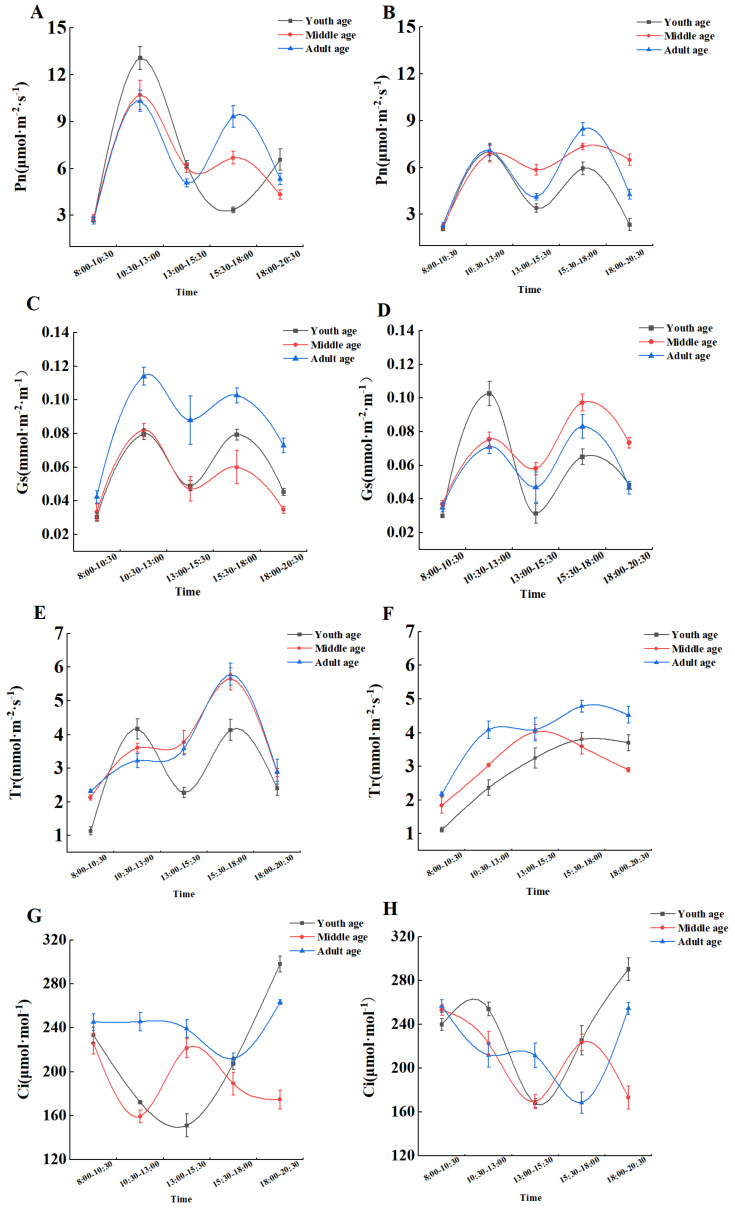
Effects of great gerbil disturbance on gas exchange parameters. (**A**) The net photosynthetic rate without gerbil disturbance. (**B**) The net photosynthetic rate under gerbil disturbance. (**C**) Stomatal conductance without gerbil disturbance. (**D**) Stomatal conductance under gerbil disturbance. (**E**) Transpiration rate without gerbil disturbance. (**F**) Transpiration rate under gerbil disturbance. (**G**) Intercellular carbon dioxide concentration without gerbil disturbance. (**H**) Intercellular carbon dioxide concentration under gerbil disturbance.

The gas exchange parameters and instantaneous water use efficiency of *H. ammodendron* at different growth stages were affected by gerbil disturbance to different degrees, and the gas exchange parameters of *H. ammodendron* at different growth stages were significantly different at different times. The net photosynthetic rate and stomatal conductance of *H. ammodendron* at each growth stage showed two peak values per day: from 10:30 a.m. to 1:00 p.m. and from 3:30 p.m. to 6:00 p.m. In the absence of gerbil interference, the net photosynthetic rate and stomatal conductivity of young, middle, and adult *H. ammodendron* were the highest from 10:30 a.m. to 1:00 p.m., and the net photosynthetic rates were 13.07 μmol·m^−2^·s^−1^, 10.7 μmol·m^−2^·s^−1^, and 10.33 μmol·m^−2^·s^−1^, respectively, while the stomatal conductance was 0.080 mmol·m^−2^·m^−1^, 0.082 mmol·m^−2^·m^−1^, and 0.114 mmol·m^−2^·m^−1^, respectively (Figure 1A,C). Under the interference of gerbils, the maximum value of the young *H. ammodendron* was still from 10:30 a.m. to 1:00 p.m., whereas the maximum values of the middle and adult *H. ammodendron* were from 3:30 p.m. to 6:00 p.m. At these times, the net photosynthetic rates of the young, middle, and adult *H. ammodendron* were 6.99 μmol·m^−2^·s^−1^, 7.34 μmol·m^−2^·s^−1^, and 8.47 μmol·m^−2^·s^−1^, respectively, while the maximum values of stomatal conductance were 0.103 mmol·m^−2^·m^−1^, 0.097 mmol·m^−2^·m^−1^, and 0.083 mmol·m^−2^·m^−1^, respectively (Figure 1B,D). The diurnal variation in stomatal conductance indicated that the disturbance of large gerbils had a greater influence on the young and middle-aged individuals than on the adults.

In the absence of the influence of great gerbils, the transpiration rate of *H. ammodendron* at each growth stage gradually increased and finally decreased between 6:00 p.m. and 10:30 p.m. Under the influence of gerbils, the transpiration rate of *H. ammodendron* at each growth stage showed two peaks: from 10:30 a.m. to 1:00 p.m. and from 3:30 p.m. to 6:00 p.m. Those of the middle and adult *H. ammodendron* plants peaked at 5.65 mmol·m^−2^·s^−1^ and 5.79 mmol·m^−2^·s^−1^ from 3:30 p.m. to 6:00 p.m., respectively, and the maximum value of the young *H. ammodendron* reached 4.17 mmol·m^−2^·s^−1^ from 10:30 a.m. to 1:00 p.m. (Figure 1E,F). The diurnal trend of the intercellular CO_2_ concentration in young and adult *H. ammodendron* before and after disturbance was essentially the same, whereas that in the middle-aged *H. ammodendron* was roughly the opposite (Figure 1G,H).

**Figure 2:**
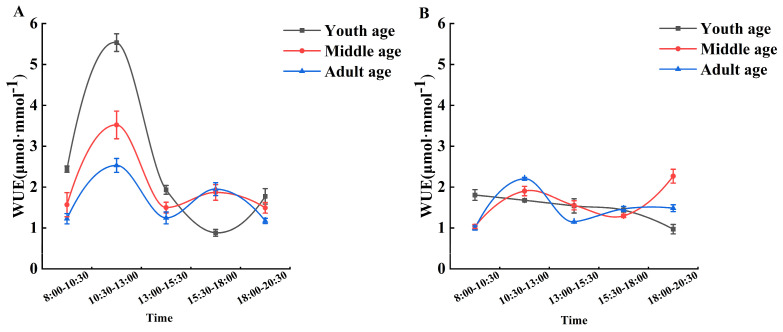
Effects of great gerbil disturbance on instantaneous water use efficiency. (**A**) The instantaneous water use efficiency without gerbil disturbance. (**B**) The instantaneous water use efficiency under gerbil disturbance.

As shown in Figure 2, the disturbance by great gerbils significantly reduced the instantaneous water use efficiency of *H. ammodendron* at all ages. The overall change range in the instantaneous water use efficiency (WUE) was 1.546–2.534 μmol·mmol^−1^ in the absence of gerbil disturbance, while the overall change range in WUE was 1.414–1.648 μmol·mmol^−1^ under the influence of gerbils. Among them, the young *H. ammodendron* changed the most after disturbance by the gerbils, whereas the influence on the adult *H. ammodendron* was small.

### 3.2. Influence of Great Gerbil Disturbance on Chlorophyll Fluorescence Parameters of H. ammodendron

**Figure 3:**
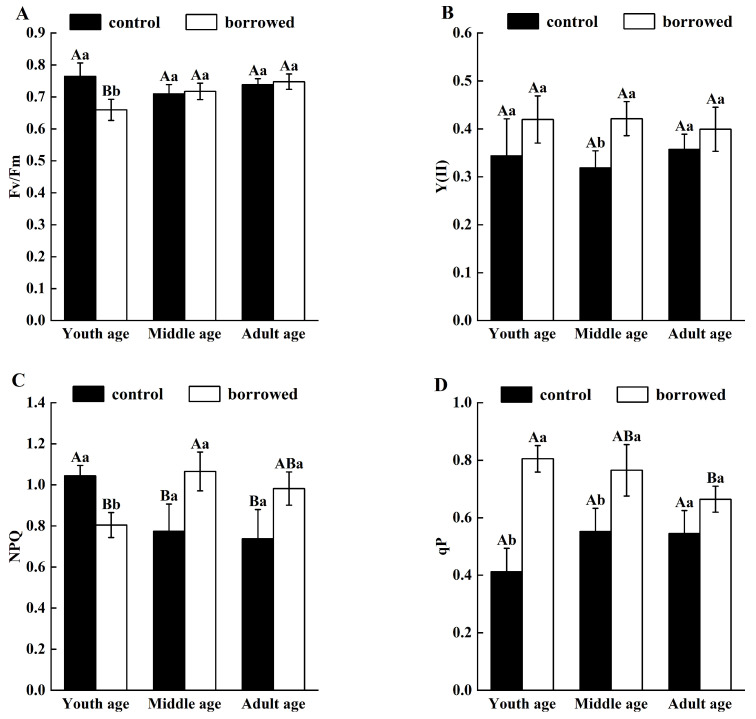
Influence of great gerbil disturbance on chlorophyll fluorescence parameters. (**A**) The maximum photochemical efficiency (Fv/Fm). (**B**) The actual photochemical efficiency (Y (II)). (**C**) The non-photochemical quenching coefficient (NPQ). (**D**) The photochemical quenching coefficient (qP). Different uppercase letters indicate that there were significant differences in the indexes of *H. ammodendron* at each growth stage (*p* < 0.5), and different lowercase letters indicate that there was a significant influence on *H. ammodendron* with or without great gerbil disturbance (*p* < 0.5).

As shown in Figure 3, under the disturbance of great gerbils, the Fv/Fm of young *H. ammodendron* decreased significantly (*p* < 0.5), but the Fv/Fm of the middle-aged and adult *H. ammodendron* increased slightly but showed no significant change. The Y(II) of *H. ammodendron* in all growth stages was higher than that in the control area, and the Y(II) of *H. ammodendron* at the young and adult ages did not increase significantly, whereas that in the middle age group did increase significantly (*p* < 0.5). The NPQ value decreased significantly in the young age group (*p* < 0.5), and there was no significant change in the middle and adult ages due to the interference of great gerbils. The qP values of the young and middle age groups were significantly increased (*p* < 0.5), but there was no significant change for the adult age group. In general, the Fv/Fm and NPQ of the young age group significantly decreased, and Y(II) and qP significantly increased in the middle age group, while there was no significant change in any indexes for the adult age group.

### 3.3. Effects of Gerbil Disturbance on Rhizosphere Soil Nutrients of H. ammodendron

**Figure 4:**
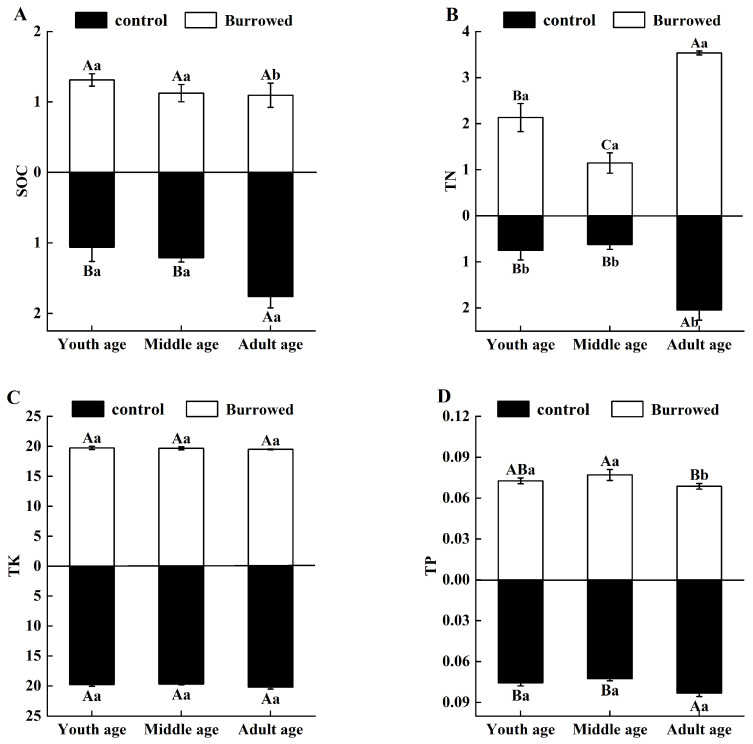
Effects of gerbil disturbance on rhizosphere soil nutrients. (**A**) Soil organic carbon (SOC). (**B**) Soil total nitrogen (TN). (**C**) Soil total potassium (TK). (**D**) Soil total phosphorus. Different uppercase letters indicate that there were significant differences in the indexes of *H. ammodendron* at each growth stage (*p* < 0.5), and different lowercase letters indicate that there was a significant influence on *H. ammodendron* with or without great gerbil disturbance (*p* < 0.5).

When there was no gerbil disturbance, the SOC, TN, TP, and TK contents in the whole rhizosphere soil of *H. ammodendron* in the three growth stages varied from 0.922 to 1.900 g·Kg^−1^, from 0.539 to 2.240 g·Kg^−1^, from 0.071 to 0.086 g·Kg^−1^, and from 19.541 to 20.512 g·Kg^−1^, respectively. The SOC, TN, TP and TK contents in the rhizosphere soil varied from 0.926 to 1.392 g·Kg^−1^, from 1.016 to 3.561 g·Kg^−1^, from 0.068 to 0.081 g·Kg^−1^, and from 19.425 to 19.992 g·Kg^−1^, respectively. The TN in the rhizosphere soil of the young, middle-aged, and adult *H. ammodendron* increased significantly (*p* < 0.05). The SOC and TP levels decreased in the adult *H. ammodendron* (Figure 4).

### 3.4. Effects of Great Gerbil Disturbance on Nutrient Uptake of H. ammodendron

**Figure 5:**
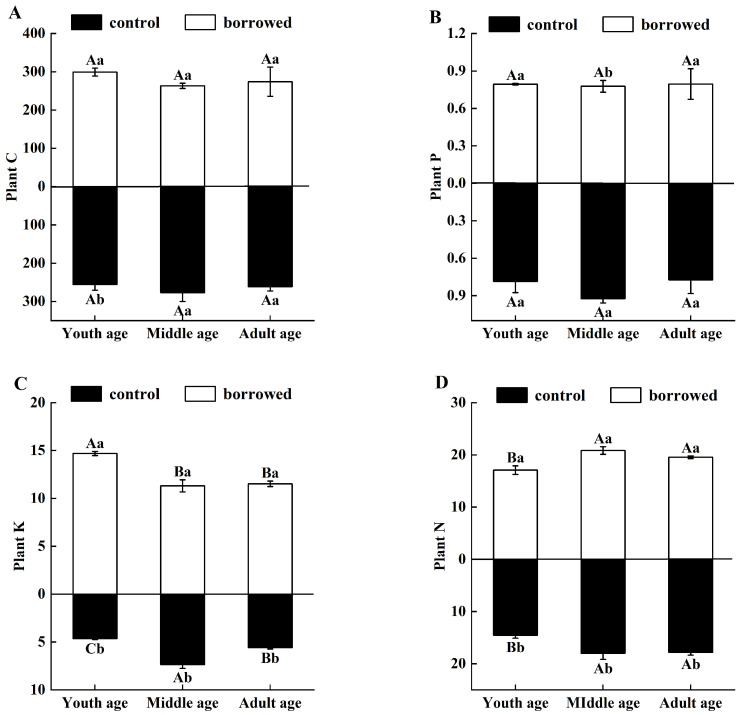
Effects of great gerbil disturbance on nutrient uptake. (**A**) Plant carbon (Plant C), (**B**) Plant phosphorus (Plant P). (**C**) Plant potassium (Plant K). (**D**) Plant nitrogen (Plant N). Different uppercase letters indicate that there were significant differences in the indexes of *H. ammodendron* at each growth stage (*p* < 0.5), and different lowercase letters indicate that there was a significant influence on *H. ammodendron* with or without great gerbil disturbance (*p* < 0.5).

In the absence of gerbil disturbance, the contents of C, N, P, and K in the assimilated branches of *H. ammodendron* in the three growth stages varied from 240 to 276.106 g·Kg^−1^, from 13.978 to 21.662 g·Kg^−1^, from 0.658 to 0.962 g·Kg^−1^, and from 4.567 to 7.805 g·Kg^−1^, respectively. Under the interference of gerbils, the changes in the C, N, P, and K contents in the assimilated branches of *H. ammodendron* were 246.729–317.647 g·Kg^−1^, 16.133–19.795 g·Kg^−1^, 0.665–0.909 g·Kg^−1^, and 10.675–14.664 g·Kg^−1^, respectively. The N and K contents of the assimilated branches of *H. ammodendron* in the young, middle, and adult growth stages significantly increased (*p* < 0.05). Among them, the C content was significantly higher in the young age group (*p* < 0.5). The P content did not change significantly in any of the three stages (Figure 5).

### 3.5. Effects of Growth Stage, Presence or Absence of Gerbils, and Their Interactions on Soil and Plant Nutrients

Changes in the nutrient content of *H. ammodendron* at different growth stages and with the disturbance of great gerbils are shown in Table 1. The SOC and TN contents in the rhizosphere of *H. ammodendron* at different growth stages were significantly different (*p* < 0.5). The N and K contents in the assimilated branches were also significantly different (*p* < 0.5). The presence or absence of gerbil disturbance had significant effects on the SOC, TN, TP, and TK contents in the rhizosphere soil of *H. ammodendron* (*p* < 0.5), whereas the study indices revealed significant effects only on K in the assimilated branches (*p* < 0.5), and the effects on other nutrients were not evident. Interaction analysis showed that the nutrient content of *H. ammodendron* at each growth stage, except for the SOC and TP, was significantly different with and without gerbil disturbance (*p* < 0.5).

### 3.6. Correlation Analysis between Nutrients and Photosynthetic Parameters

**Figure 6:**
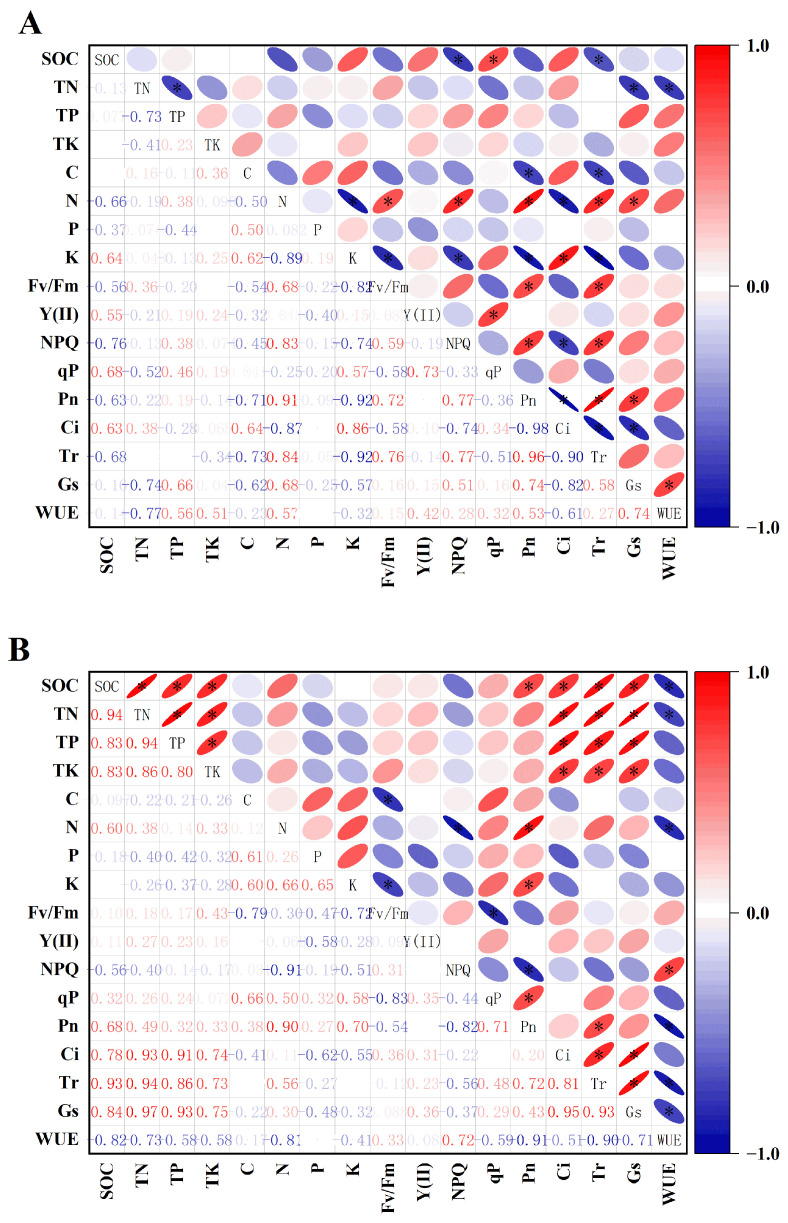
Pearson correlation coefficient heat map of nutrient and photosynthetic parameters. Note: “*” indicates that there was a significant correlation between indicators. (**A**) Rat hole area (with great gerbil disturbance). (**B**) Control area (without great gerbil disturbance).

As shown in Figure 6A, in the rat hole region, the SOC and qP; Fv/Fm, NPQ, Pn, Tr, Gs, and N; Fv/Fm, NPQ, and Pn; K and Ci; and Fv/Fm, NPQ, and Tr showed significant positive correlations. The K and Fv/Fm; SOC, K, and NPQ; C, K, and Pn; NPQ and Ci; SOC, TN, C, K, and Tr; and Gs, WUE, and TN showed significant negative correlations.

In the control area, all soil nutrients were positively correlated with the gas exchange parameters except for the Pn. The Pn and SOC; K, P, qP, and Pn; and NPQ and WUE showed significant positive correlations. The WUE was significantly negatively correlated with the C, K, and Fv/Fm; N, Pn, and NPQ; and SOC, TN, N, and other gas exchange parameters, except the Ci (Figure 6B). In general, there was a positive correlation between the soil nutrients and gas exchange parameters in the rhizosphere of *H. ammodendron* without gerbil disturbance. However, this characteristic changed significantly after gerbil disturbance.

## 4. Discussion

### 4.1. Changes in Photosynthetic Characteristics and Instantaneous Water Use Efficiency of H. ammodendron

The response of *H. ammodendron* to great gerbil disturbance was investigated by observing changes in the photosynthetic characteristics and soil nutrients in the assimilating branches and rhizospheres at different growth stages. Great gerbils mainly affect the growth and development of *H. ammondendron* in two ways.: the first is direct physical damage, such as nibbling on the assimilated branches of *H. ammodendron*, which reduces the leaf area of the plant for photosynthesis, and the second way is through indirect ecological effects, which affect the photosynthetic characteristics and instantaneous water use efficiency of *H. ammodendron* by changing the soil nutrient status. Photosynthesis is an important process for plant growth and reproduction and is affected by drought, high temperatures, and other factors [34]. In this study, it was found that diurnal variation in the net photosynthetic rate of *H. ammodendron* at different ages in the two plots showed a “bimodal pattern”, which is consistent with previous studies [35]. This “bimodal” diurnal variation pattern reflects the adaptation strategy of *H. ammodendron* to extreme environmental conditions (such as high temperatures and strong light at noon) [36]. Therefore, when there was no great gerbil disturbance, the Pn, WUE and Gs reached the maximum value at each growth stage from 10:30 a.m. to 1:00 p.m., and the net photosynthetic rate decreased from 1:00 p.m. to 3:30 p.m. to make the photosynthetic lunch break appear in *H. ammodendron*. The Pn, WUE, and Gs were reduced to reduce water loss and prevent damage to the photosynthetic mechanism, and they gradually recovered from 3:30 p.m. to 6:00 p.m. This phenomenon is found in many desert plants, such as *Tetraena mongolica*, *Sarcozygium xanthoxylon*, *Ammopiptanthus mongolicus*, and *Nitraria tangutorum* [37]. In the case of great gerbil disturbance, the Pn and Gs still showed the same trend as those in the control area, but the disturbance of great gerbils significantly reduced the Pn of *H. ammodendron* in each growth stage from 10:30 a.m. to 1:00 p.m., and the Pn did not change significantly compared with the control area in other time periods. Therefore, in general, the disturbance of the great gerbil reduced the Pn of *H. ammodendron* at all growth stages, which may be because the disturbance of the great gerbil affected the nutrient absorption of the root system of *H. ammodendron*, which reduced the nutrient intake capacity of the leaves, thereby reducing the Pn of *H. ammodendron*. Compared with other indexes measured in this study, the diurnal variation in the Tr after the disturbance of great gerbils showed a steady upward trend and then decreased, which may be due to the mechanical damage caused by the great gerbil and enhancement of the water absorption capacity in order to maintain the growth of the plant as the temperature increased during the day.

In addition, we explored the changes in the chlorophyll fluorescence parameters (such as the Fv/Fm, Y(II), NPQ, and qP) under the interference of gerbils, which reflect the changes in the photoconversion efficiency of plant photosynthetic institutions under environmental stress [38]. In this study, it was found that the disturbance of great gerbils had a significant effect on the chlorophyll fluorescence parameters of young *H. ammodendron*, and the disturbance of great gerbils reduced the Fv/Fm and NPQ of young *H. ammodendron*, indicating that the disturbance of great gerbils limited the photosynthetic activity of young *H. ammodendron*, and its photoprotection ability was reduced. This may be due to the fact that young *H. ammodendron* are not fully adapted to the external environment, and thus they are more sensitive to the disturbance of great gerbils and have a weaker ability to adapt to the external environment. There was no significant difference in the chlorophyll fluorescence parameters of the adult *H. ammodendron* before and after the disturbance of great gerbils, indicating that during the long-term coexistence of *H. ammodendron* and great gerbils, it has developed certain ecological adaptations to the interference of great gerbils. The higher Y(II) and qP of the middle-aged *H. ammodendron* under gerbil disturbance indicate that soil drought and gerbil disturbance opened the PS II reaction center, enhanced proton flow, optimized light energy distribution, and improved photochemical reaction efficiency to cope with external biotic and abiotic stresses [39,40]. Therefore, in terms of light energy use efficiency, the disturbance of gerbils makes young *H. ammodendron* vulnerable to damage.

The water use efficiency (WUE) of plants reflects the relationship between the consumption of water and the dry matter of plants, and it is an important indicator of plant drought tolerance [41]. Studies have found that perennial shrubs increase their WUE when the soil water content is dry to moderate. However, when the soil water content is extremely low, stomatal conductance eventually becomes conservative and stable, and the WUE begins to decline again [42]. In this study, the WUE performance of *H. ammodendron* was higher in the absence of interference from gerbils, because *H. ammodendron* was able to maintain a high net photosynthetic rate (Pn) and control transpiration rate (Tr) at a relatively low level. In contrast, the disturbance of great gerbils led to a decrease in the Pn and Gs of *H. ammodendron* by directly damaging and changing soil nutrients, whereas the Tr did not change significantly, resulting in a decrease in the WUE. This may be due to the decrease in the photosynthetic area of plants caused by the feeding of assimilated branches of *H. ammodendron* to great gerbils. Correlation analysis also showed that there was a positive correlation between the WUE and Pn of *H. ammodendron* under the disturbance of giant gerbils, as well as a significant positive correlation between the WUE and Pn and Gs. This further confirmed that giant gerbils had a negative impact on the growth and development of *H. ammodendron* by affecting its photosynthesis and water use efficiency.

In conclusion, the present study showed that gerbil interference changes the photosynthetic characteristics and instantaneous water use efficiency of *H. ammodendron* through direct and indirect effects, thereby affecting the growth and ecological adaptation of *H. ammodendron*. These findings provide an important scientific basis for understanding how *H. ammodendron* survives and adapts to long-term coexistence with great gerbils.

### 4.2. Changes in Assimilated Branch Nutrients and Rhizosphere Soil Nutrients of H. ammodendron

Through its ecological behaviors, such as digging and feeding, gerbil disturbances significantly changed the nutrient status of the rhizosphere soil and nutrient contents in the assimilated branches of *H. ammodendron*. In this study, we found that the disturbance of great gerbils significantly increased the total nitrogen (TN) content in rhizosphere soil nutrients at each growth stage, which may be related to the fact that the digging behavior of great gerbils promotes soil gas exchange and nutrient cycling. Because the gerbil digging process fully mixed the soil and increased the internal homogeneity of the soil, the excretion of feces and other activities increased the TN content in the soil [43]. In this study, there were significant differences in the TN content in the rhizosphere soil of *H. ammodendron* at different growth stages, and the TN content in the rhizosphere soil of *H. ammodendron* was significantly higher than those of the young and adult age groups, which may be due to the fact that compared with the young and middle age groups, giant gerbils mainly nested in the roots of *H. ammodendron* and were prone to accumulating urine and feces, and thus the TN in the rhizosphere soil of adult *H. ammodendron* was significantly higher than those of the young and adult age groups. In contrast, the SOC in the rhizosphere soil nutrients decreased in the adults, while there was no significant change in the SOC between the young and middle age groups. In nature, organic soil originates from plant residue and organisms that grow on the soil’s surface [44]. Studies have shown that strong disturbances caused by gerbils can reduce the SOC content of soil nutrients [31]. Owing to the interference of great gerbils, few other plants grew in the rat hole area, with only a few short-lived plants and rather few plant residues [31]. Moreover, we found in our study that most of the great gerbils dug burros in the rhizospheres of adult *H. ammodendron*, and thus SOC reduction may be due to the fact that the digging activities of the great gerbils reduce the accumulation of plant residues in the soil and reduce the source of organic matter. In this study, the TN content of the rhizosphere soil nutrients increased at each growth stage, and the SOC content of the rhizosphere soil nutrients decreased in adults. However, it is still unknown whether great gerbils perform the same disturbance to *H. ammodendron* in different seasons or what the effects of the excreta conposition on soil nutrients are, which need to be studied further.

In addition, the interference of giant gerbils significantly increased the contents of nitrogen (N) and potassium (K) in the assimilated branches of *H. ammodendron* at the young, middle, and adult growth stages, which may be related to the enhanced absorption and accumulation of nutrients by plants to compensate for the eaten parts. As giant gerbils eat the assimilated branches of *H. ammodendron* for a long time, the plants maintain their growth in response to external interference, strengthening the absorption and accumulation of nutrients in the plants. Some studies have suggested that the content and activity of a plant’s photosynthetic enzymes may increase to compensate for the damage to photosynthetic organs under stress conditions [45]. At the same time, it may also be related to the enhancement of plant food resistance. Studies have shown that to improve plant resistance to external herbivores, the content of secondary metabolites secreted by plants increases, thus increasing the demand for nutrients [46]. In addition, the new plant tissues increase under the feeding of external herbivores. There may be an increase in nutrient concentrations compared with the aging tissues of plants in the control area [45]. At the same time, this study found that the N and K contents of the assimilated branches of young *H. ammodendron* were lower than those of the middle- and adult-aged plants without gerbil interference because the photosynthesis area, root development, and environmental adaptability of the young plants were lower than those of the middle- and adult-aged plants, and thus the N and K contents in the assimilated branches were relatively low. When there was disturbance by giant gerbils, the K content in the assimilated branches of young *H. ammodendron* was higher than those of the middle- and adult-aged *H. ammodendron*, and the N content was lower than those of the middle- and adult-aged *H. ammodendron* because the main source of K in plants is root absorption from the soil [47]. Compared with the middle- and adult-aged *H. ammodendron*, the young *H. ammodendron* was more sensitive to external disturbances and more susceptible to external disturbances. In addition, there was no significant difference in the soil TK content at different growth stages under the disturbance of giant gerbils, and the absorption capacity of K was enhanced in order to maintain the nutrient balance in the body of *H. ammodendron* and maintain its own growth. Compared with the young *H. ammodendron*, the gnawing of middle- and adult-aged *H. ammodendron* was more severe, and the middle- and adult-aged *H*. *ammodendron* compensated for the feeding of the gerbils and enhanced the absorption of N when the soil’s TN was sufficient.

## 5. Conclusions

The disturbance behavior of the gerbils had a significant impact on *H. ammodendron* and its growth environment, which not only changed the soil nutrient state but also the photosynthesis capacity of *H. ammodendron*. In terms of soil and plant nutrients, under the disturbance of gerbils, the TN in the rhizosphere soil nutrients increased significantly at each growth stage, and the SOC in the rhizosphere soil nutrients decreased in adults. The N and K contents of the plant nutrients increased significantly at each growth stage, whereas the P content showed no significant change. In terms of the plant’s photosynthetic properties, the net photosynthetic rate and instantaneous water use efficiency were lower than those in the control area. At the same time, the Fv/Fm and NPQ values of the young *H. ammodendron* were also lower than those in the control area. The results of this study revealed that the disturbance of great gerbils significantly increased the TN content in the rhizosphere soil nutrients and the N and K contents in plant nutrients and decreased the photosynthetic capacity. The effect on the photosynthesis of young *H.ammodendron* was the most obvious one. These findings reveal the complex ecological interaction mechanism between gerbils and *H. ammodendron* and have important scientific significance for understanding plant environmental adaptation strategies in arid ecosystems.

## Figures and Tables

**Table 1:** Two-factor variance analysis of soil and plant nutrients.

		Soil Nutrients				Plant Nutrients			
		SOC	TN	TP	TK	C	N	P	K
Growth stage	F	6.139	138.574	0.823	0.845	0.371	39.817	1.274	17.120
	P	0.015	<0.0001	0.462	0.453	0.698	<0.0001	0.315	<0.0001
Presence or absence of gerbils	F	6.101	138.895	14.159	4.904	2.226	1.840	1.064	1703.790
	P	0.029	<0.0001	0.003	0.047	0.162	0.200	0.323	<0.0001
Interactions	F	15.806	10.161	21.938	4.102	2.395	23.409	2.052	124.152
	P	<0.0001	0.003	<0.0001	0.044	0.092	<0.0001	0.171	<0.0001

## Data Availability

The raw data supporting the conclusions of this article will be made available upon request to the corresponding authors.
